# The process using a synthetic library that generates multiple diverse human single domain antibodies

**DOI:** 10.1093/abt/tbae020

**Published:** 2024-08-03

**Authors:** Mark A Tornetta, Brian P Whitaker, Olivia M Cantwell, Eileen D Pisors, Lu Han, Maria P MacWilliams, Hao Jiang, Fulai Zhou, Mark L Chiu

**Affiliations:** Biologics Discovery Department, Tavotek Biotherapeutics, 727 Norristown Road, Spring house Innovation Park, Building 3, Suite 101, Lower Gywnedd, PA 19002, United States; Biologics Discovery Department, Tavotek Biotherapeutics, 727 Norristown Road, Spring house Innovation Park, Building 3, Suite 101, Lower Gywnedd, PA 19002, United States; Biologics Discovery Department, Tavotek Biotherapeutics, 727 Norristown Road, Spring house Innovation Park, Building 3, Suite 101, Lower Gywnedd, PA 19002, United States; Biologics Discovery Department, Tavotek Biotherapeutics, 727 Norristown Road, Spring house Innovation Park, Building 3, Suite 101, Lower Gywnedd, PA 19002, United States; Biologics Discovery Department, Tavotek Biotherapeutics, Building C2, Suzhou Biomedical Industrial Park, Suzhou, Jiang Su 215000, China; Biologics Discovery Department, Tavotek Biotherapeutics, 727 Norristown Road, Spring house Innovation Park, Building 3, Suite 101, Lower Gywnedd, PA 19002, United States; Biologics Discovery Department, Tavotek Biotherapeutics, Building C2, Suzhou Biomedical Industrial Park, Suzhou, Jiang Su 215000, China; Biologics Discovery Department, Tavotek Biotherapeutics, Building C2, Suzhou Biomedical Industrial Park, Suzhou, Jiang Su 215000, China; Biologics Discovery Department, Tavotek Biotherapeutics, 727 Norristown Road, Spring house Innovation Park, Building 3, Suite 101, Lower Gywnedd, PA 19002, United States; Biologics Discovery Department, Tavotek Biotherapeutics, Building C2, Suzhou Biomedical Industrial Park, Suzhou, Jiang Su 215000, China

**Keywords:** binning, biopanning, cell binding, epitopes, internalization, nanobody, paratopes, phage display, single domain antibody, variable heavy chain

## Abstract

**Background:**

Single domain antibodies (sdAbs) possess unique characteristics that make them highly effective for developing complex therapeutics.

**Methods:**

Our process uses a fully synthetic phage display library to generate single domain antibodies that can bind to disease relevant antigen conformations. A human IGHV3 family scaffold makes up the phage display libraries, and these VHO libraries are applied to diverse phage biopannings against target antigens. After NGS processing, unique VHOs undergo automated cloning into expression constructs followed by transfections and purifications. Binding assays were used to determine VHO binding behaviors to the target proteins. Additional VHO interactions are measured against endogenous targets on cells by way of flow cytometry, cell internalization, and activation assays.

**Results:**

We show that a fully synthetic phage display library can generate VHOs that bind to disease relevant antigen conformations. The diverse biopanning methods and processing of next-generation sequencing generated many VHO paratopes. These different VHO sequences can be expressed as Fc fusion proteins. Various screening assays resulted in VHOs representing different epitopes or activities. During the hit evaluation, we demonstrate how screening can identify distinct VHO activities that have been used to generate differentiated drug molecules in various bispecific and multispecific antibody formats.

**Conclusion:**

We demonstrate how screening can identify distinct VHO activities that have been used to generate differentiated drug molecules in various bispecific and multispecific antibody formats.

## Introduction

**Figure 1:**
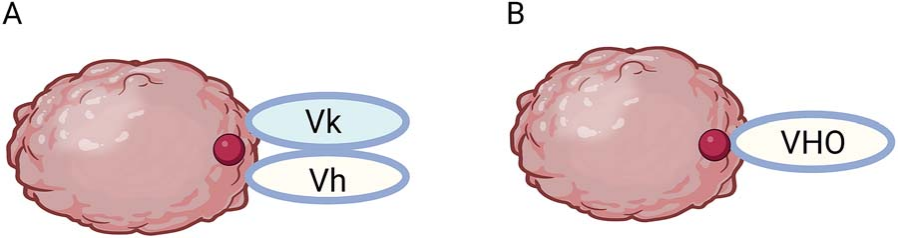
Schematic representing (A) an IgG or Fab or scFv paratope interacting to a target protein with an average surface area of 834 Å2 and (B) a sdAb or VHO paratope interacting to the same target protein with an average surface area of 768 Å2.

Structural differences between antibody formats can impact their overall functionality when developing biologics for various applications such as therapeutics, biomarkers, diagnostics, and validation of biological mechanisms [1]. Figure 1 illustrates a comparison of the binding interactions of an IgG Fab (a) and a sdAb (b) to an epitope on a protein target. A human IgG antibody has an Fc domain and a 50 kDa 4 nm × 5 nm sized Fab domain that comprises two disulfide-linked heavy and light chain polypeptides with 50 kDa a calculated binding surface area of 600–900 square angstroms (Å^2^) [2]. A single domain antibody, also called a VHH, consists of a 12–15 kDa 2.5 × 4 nm sized heavy chain variable domain with a calculated binding surface area of 600–800 Å^2^ [2]. The larger size of an IgG Fab can have structural steric hindrances that constrain the range of binding epitopes on a target. However, the smaller sdAb paratope has a higher chance of generating candidates with a greater diversity of epitopes. In addition, the smaller paratopes of sdAb can allow for more precise engagement of the target molecules [3–6].

Single domain antibodies possess several advantageous properties in comparison to conventional Ig antibodies, scFvs, and Fab formats. This makes sdAbs more suitable for both diagnostic and therapeutic applications. Compared with Ig antibodies, sdAbs have longer CD3 lengths that can result in a greater diversity of paratopes to enable recognition of a wider range of epitopes.

Single domain antibodies, with a simpler heavy chain-only structure, can refold more easily after exposure to changing temperatures, as compared with other Ig formats [7]. They can refold at either lower or higher temperatures, and their structure remains intact, allowing them to bind to and detach from targets without encountering issues such as aggregation or denaturation due to multi-subunit interactions between the heavy and light chains of IgG molecules [1]. Hydrophobic interactions between the heavy and light variable regions drive the overall structures of scFv and Fab molecules. In the absence of stabilizing domains, such as Fc and CH1 and CL domains for scFv, these hydrophobic interactions of the variable regions can cause instability and limit solubility, leading to aggregation [7].

Single domain antibodies of camelid origin have more hydrophilic variable region frameworks compared with conventional IgG molecules [8]. This hydrophilicity allows sdAbs to tolerate a wide range of pH conditions [9], including harsh acidic conditions [10]. This property of sdAbs could be advantageous for therapeutic applications where the disease state is in an acidic environment, such as acidosis [11–13].

The physical properties of sdAbs, such as their small size, rigid structure, greater tolerance to thermal fluctuation, and stability at lower pH, make them superior candidates for efficient manufacturing [14]. However, the use of camelid-based sdAbs generated by immunization poses the possibility of immune response, so humanization techniques are usually applied to minimize such reactions by patients [15]. To manage the development of potential immune responses, both synthetic and semi-synthetic phage display that libraries constructed onto humanized camelid frameworks have been developed [16–18]. Nonetheless, such recombinant humanized sdAbs can have instability characteristics like that of the Fab and scFv-based molecules.

The traditional method of obtaining biomedically relevant sdAbs through immunization and screening of animals (alpaca, llama, camels) can be challenging. These animals produce heavy chain-only antibodies in response to the immunization. While capturing the proper pairing of the heavy and light chains for Fab or scFv from an immunized source [19] is effective, the selection of the VHH still must deal with the challenges of antigen tolerance, PCR bias, and the challenge of posttranslational differences between natural B-cell and recombinant host cell expression [20]. In addition, the immunization regime and screening campaign require increased labor, time, and cost when compared with a phage library selection [19,21,22].

**Figure 2:**
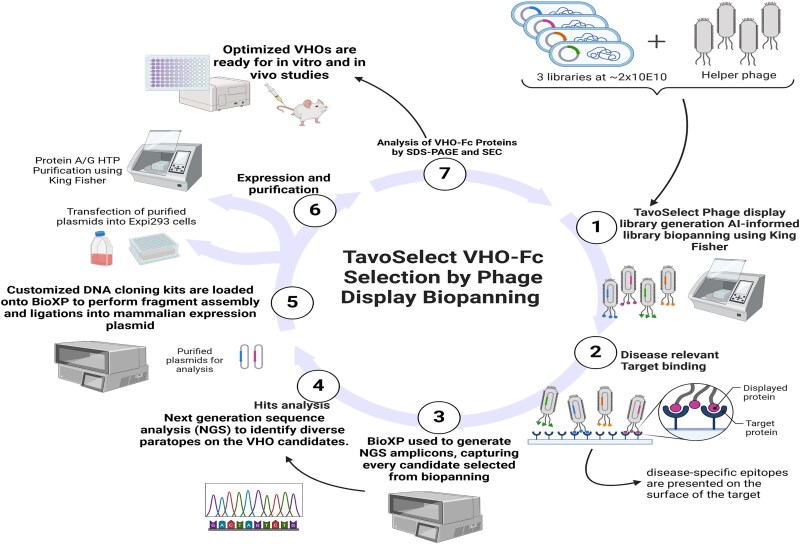
A visual representation of the TavoSelect process to generate VHO-Fc proteins.

Previously, we successfully generated antibodies from fully synthetic Fab libraries [23]. We have expanded this process to develop a fully human synthetic sdAb phage display library termed the VHO antibody library. To facilitate the creation of VHO-based biologics with great efficiency, a process called TavoSelect was designed (Fig. 2). The TavoSelect VHO platform starts with fully synthetic human single domain scaffold libraries applied into diverse phage display selection processes to generate candidates against varying disease target formats (Fig. 2, steps 1, 2). Selected VHOs are then captured by NGS to distinguish a diverse array of paratopes that contain minimal to no development risks (Fig. 2, step 3). We carefully screen VHOs and fuse them to a human IgG Fc through automated cloning, using the BioXP™ instrument (Fig. 2, step 5). This process overcomes the poor PK of native sdAbs that are used in animal studies [24]. Here, we also describe our semi-high throughput expression and purification processes to generate VHO proteins (Fig. 2, step 6). Each protein undergoes a comprehensive assessment of binding assays that confirm the diversity of activity bins (Fig. 2, step 7). VHOs representing separate activity bins are paired with VHOs with different epitopes to evaluate avidity effects that translate into improved target engagement interactions. This TavoSelect platform process generates diverse sets of VHOs to enable the engineering of differentiated molecules for specific on-disease target activity.

## Materials and methods

Bacterial media and agar plates were obtained from Teknova or KD Medical. Tissue culture products were purchased from ThermoFisher Scientific, Invitrogen, Gibco, and Sigma. Cell lines were obtained from a cancer cell line (ATCC) and Lucigen. Telesis Bio, Gene Art, IDT DNA, Genewiz, and GenScript performed gene synthesis for the phage library and expression constructs for VHO-Fc protein production. Target proteins were purchased from ACRO Biosystems, Sino Biological, and R&D Systems of Bio-Techne. Finally, all other reagents and disposables were purchased from Thermo Fisher Scientific.

### VHO scaffold phage display libraries

All references of the sequences use the single letter amino acid code. The libraries were created using the human IGHV3 family based on IMGT [25] classification. The amino acid combinations of CDR1 and CDR2 were limited to less than 10^5^ diversities incorporating all residues except C, as per the research conducted by M. Dondelinger *et al.* (2018) and L. Shi *et al.* (2010) [23,26]. The CDR3 pool was made by oligonucleotide fragments of eight different lengths, randomized at each position incorporating all residues except C, M, N, for one design and minimize C, M, N with another design to generate a diversity greater than 10^14^. The CDR definitions were based on Kabat classification [27]. Telesis Bio and Gene Art-ThermoFisher Scientific carried out gene synthesis. Linear synthesized libraries were created via traditional restriction enzyme and DNA ligation methodologies. Each of the eight CDR3 length libraries was ligated into a dual-function bacterial expression phagemid using a method like that described by L. Shi *et al.* (2010). Electroporation (BTX) was used to transform MC1061 F′ cells (Lucigen). The transformed libraries were propagated, and the cultures were subsequently infected by the VCSM13 filamentous bacteriophage, as recommended by the manufacturer (Agilent Technologies). The infected cultures were expanded and induced to produce recombinant VHO-pIX fusion proteins displayed on phage particles. After 12–16 h of incubation, the centrifuged cultures were collected in fresh tubes for PEG/NaCl precipitation. The precipitated phage library supernatants were centrifuged, and the pellets were resuspended in 1× PBS and stored at −80°C.

### Automated phage biopanning to generate VHOs

The automated, high-throughput phage biopanning was performed using the KingFisher Apex system from ThermoFisher Scientific. Streptavidin-coated magnetic beads (Invitrogen™ Dynabeads™ M-280 Streptavidin) and/or nickel-coated beads (Thermo Fisher™ NiNTA magnetic beads) were used to capture biotinylated or polyhistidine tagged target antigen, respectively. The phage library pool was incubated with the target protein-coated beads, and the KingFisher magnet was used to collect the target-coated magnetic beads along with any VHO phage that had bound. The residual unbound phage was removed in subsequent wash steps. The bound phage was captured by adding log phase MC1061 F′ cells and subjected to an additional two to three biopanning rounds of amplified phage binders. To eliminate non-specific VHO-phage binding activity, at least, one round of biopanning was performed using only the streptavidin magnetic beads without target antigen present. To conduct a biopanning campaign with maximum diversity, the VHO phage library was used to target the presented format using different methods such as human, mouse, cynomolgus full-length proteins, extracellular domains (ECDs), mammalian cells displaying the target, etc. The biopanning was also conducted under relevant disease conditions such as an acidic pH of 6.0 commonly found in tumor environments. After the last round of selection, the phage pool underwent DNA extraction. The extracted DNA was then used as a template to create NGS libraries using the BioXP™ system from Telesis Bio. To capture all three CDR sequences, primers in Frameworks 1 and 4 were utilized for creating NGS libraries. The sequencing was carried out using either a MiSeq or NextGen (Illumina) platform. The sequence data were processed using the Pipe Bio software from PipeBio, Inc.

### VHO-Fc expression plasmid construction via automated cloning

The sequences acquired from the NGS processing software were used to create customized DNA cloning kits from Telesis Bio. These cloning kits were loaded onto the BioXP™ platform to perform fragment assembly and ligation into a pcDNA3.4-based mammalian expression plasmid (Thermo Fisher). The VHO portion was fused at the c-terminal end to a G_4_S linker fused upstream of the hinge, including the cysteines of a human IgG1 Fc. The resulting plasmids were transformed into TOP10 *Escherichia coli* cells (Invitrogen). Single colonies from each transformation were grown for DNA preparation. The DNA plasmid preparations were sequenced by Sanger (Azenta Life Sciences or Eton Bioscience, Inc.) to ensure that the correct DNA plasmids obtained an in-frame signal sequence and VHO fused to human IgG1 Fc (VHO-Fc). The same cloning techniques were used for the construction of the dual VHO-Fc constructs, where a G_4_S linker is present between the N-terminal– and C-terminal–oriented VHOs. Another G_4_S linker was located between the tandem VHOs and the human IgG1 Fc. The bispecific molecules were encoded by genes that have either a single or dual VHO fusion to a human IgG1 as one arm and the CD3 agonist scFv fused to a human IgG1 Fc as the other arm where each contained control arm exchange mutations [28].

### Transfections and automated VHO-Fc purifications

The VHO-Fc plasmids were transfected using the Expi-293 transient expression kit from Gibco™. After 5 days of incubation at 37°C and 8% CO_2_, the media supernatants were harvested. These supernatants were then processed using magnetic beads coated with protein A or G from Promega in 24 or 96 well plates. The KingFisher was used for automated binding, washing, and elution, followed by manual neutralization of the eluted material. The purified VHO-Fc molecules were analyzed by SDS-PAGE with 1 μg of purified protein and SeeBlue™ Plus2 Pre-stained Protein Standard (Invitrogen™) on a 4%–12% gradient Bis-Tris polyacrylamide gel (Invitrogen™). Protein bands were visualized using SafeStain (Invitrogen™). The VHO-Fc purified proteins were also analyzed by SEC using an HPLC Column [TSKgel G3000SWXL, Silica, 5 μm, 7.8 mm × 30 cm (pn:0008541)]. The samples were injected neat, with the volumes adjusted for 30-μg protein load. The running buffer was 1× PBS. The instrument settings were as follows: wavelengths 280 and 214 nm; bandwidth: 4 nm; reference wavelength: off; flow rate: 0.9 or 0.8 mL/min; pressure limit: 70 bar; column temperature: 25°C; and autosampler temperature: 4°C. For controlled arm exchange [28], an equimolar mixture of the two arms was mediated with 2-MEA.

### Biolayer interferometry kinetics

Binding kinetics were performed on a Gator BLI instrument following vendor-recommended standard operating protocols, as described by [29]. The human, mouse, and cynomolgus monkey target proteins were captured to Ni-NTA sensor probes (Gator Bio) if they were HIS tagged and captured to streptavidin sensor probes if the target proteins were biotinylated. The sensor loading step was performed at pH 7–7.4 using 1× PBS. Before the association phase, a baseline step was performed to remove excess target protein. This step was carried out using either 1× PBS pH 6.0 or 1× PBS pH 7–7.4. The 1× PBS pH 6.0 was used to mimic the slightly acidic TME. The association and dissociation phases were run using the same pH as during the baseline step. VHO-Fcs were added as the analytes to measure binding kinetics, and the final kinetic rates of binding were calculated using the Gator Bio analysis software. Positive BLI binding activity was scored according to the response signal of >0.2 nm at the point of equilibrium (no further change in BLI response) during the analyte association phase. To establish specificity and/or background, both a negative target protein and a negative VHO-Fc were used.

### ELISA binding

The target protein was applied to Maxisorp plates (Nunc). This was done either directly or through streptavidin interaction with the biotinylated target. If available, human, mouse, or cynomolgus orthologs were applied to multiple wells of a Maxisorp plate. The plate was blocked with either nonfat milk dissolved in the assay buffer (1× PBS, 1× PBS-Tween20) or SuperBlock (Thermo Fisher). VHO-Fcs were added in serial concentrations ranging from 100 nM to less than 10 pM and incubated for an hour with shaking. VHO-Fc binding was detected by an horseradish peroxidase (HRP) conjugated anti-IgG1 Fc secondary antibody (Jackson Immunologicals) followed by an OPD substrate or a chemiluminescence substrate (Thermo Fisher, Seracare). The plates were read on the Molecular Devices SpectraMax i3× and the results were analyzed with SoftmaxPro (Molecular Devices), Prizm (GraphPad), or Excel software (Microsoft 365). Negative controls were VHO molecules specific to other target proteins, and positive controls were well-characterized target-specific IgGs. Positive binding was defined by having a signal greater than three times that of negative controls at a concentration no greater than 10 nM.

### Cancer cell binding

ATCC was used to test VHOs that target a specific protein. A range of 5 × 10^3^ to 10^4^ cells were added to each of the 96 wells of a microtiter plate, and cells were pre-incubated with an FcR blocking reagent (BD Biosciences). The VHOs were added in serial concentrations from 100 nM to less than 100 pM in either 1× PBS or flow cytometry buffer (BD Biosciences). After incubation for 1–2 h, cells were washed at least once with a flow cytometry buffer. A secondary antibody specific for IgG1 Fc domain (Jackson Immunological) labeled with Alexafluor647 or FITC was added at a concentration that provided an optimal signal-to-noise ratio (ratio > 5) in flow cytometry buffer. The plates were washed again and resuspended in Read Solution (Thermo Fisher) or flow cytometry buffer (BD Biosciences) before being read in a flow cytometer (Attune from Thermo Fisher). All steps were performed at 4°C. FlowJo software v10.8 was used for data analyses. Negative controls were VHOs specific to other target proteins, and positive controls were well-characterized target-specific IgGs. Positive binding was defined as having a signal greater than three times that of negative controls at a concentration no greater than 10 nM.

### Cell internalization

MKN45 cancer cells (ATCC) that naturally express the target were added to a 96-well microtiter plate at 5 × 10^4^ to 10^5^ cells. VHO-Fcs were tagged with pH-sensitive fluorophore pHrodo reagents according to the vendor’s instructions (Thermo Fisher). The cells were then incubated with pHrodo labeled VHO-Fcs at 10 and 1 μg/mL for 1–24 h in a tissue culture incubator at 37°C and 5% CO2. After incubation, the cells were washed, and each sample-well was imaged using a fluorescent microscope to determine the intensity of the green color and its location in the cells. Imaging parameters and cell preparation were obtained prior to imaging. Negative controls consisted of VHOs specific to another target protein, whereas positive controls consisted of well-characterized target-specific IgGs.

### T cell activation bioassay

Jurkat-NFAT effector cells (Vazyme, DD1302) were used to test T cell activation activity according to the manufacturer’s protocol. First, U-87 MG target cells (ATCC) were seeded into a 96-well white plate and left to incubate overnight. The next day, the medium was removed, and Jurkat-NFAT effector cells were added to each well with the target cells at a ratio of 6:1. Antibodies with a three-fold dilution series were also added to each well. The plate was then incubated at 37°C with 5% CO_2_ for 6 h, which activated the Jurkat T cells. After incubation, the plate was brought to room temperature and left for 10 min. Then, Bio-Lite luciferase assay substrate (Vazyme, DD1201-01) was added to each well in the dark and left for another 10 min to stabilize the signal. Finally, the luminescence signal was measured using a Tecan multi-plate reader.

## Results

### VHO phage library

The candidate pools for each target were generated from fully synthetic VHO phage display libraries that were designed to ensure diversity in the library design and to capture screening diversity in our resulting candidates. One set of the synthetic library diversity used the trimer oligo technology [30] to ensure minimal posttranslational risks and minimize unintended presence of stop codons [22]. This library was designed to be as simple as possible encompassing the codons for all protein residues (except for those corresponding to M, N, C, and stop codon) that were distributed across CDR3 regions found between the flanking motifs of YYCA and FDYW of the human heavy variable subfamily IGHV3-23 (IMGT). The library design diversity for the CDR3 length of 10–17 residues was between 1.88 × 10^12^ and 1.88 × 10^19^. The synthetic library was complemented by the additional use of the Gibson technology to improve the efficiency of NNM (where N = A/C/G/T and M = A/C) mutagenesis coding to most residues within the CDR3 regions. The design diversity for these CDR3 length of 10–17 residues libraries was between 2.21 × 10^11^ and 2.21 × 10^17^. The transformation efficiencies for each library and each CDR3 length ranged from 5 × 10^8^ to 2 × 10^9^. By combining the two synthetic phage library designs, we created a phage display library of 2 × 10^10^ size. NGS analyses on >1 million pooled CDR3 sequences confirmed >95% accuracy of the mutational design without any enrichment of individual sequences.

### Generating VHOs from biopanning and NGS

A fully synthetic library was used to generate various VHO domains that underwent targeted biopanning processes, resulting in the selection of candidates with diverse paratopes. To capture as many relevant disease epitopes as possible, the target was presented in various states. When dealing with cancer targets, biopanning was carried out at both pH 7.4 and a solid tumor-relevant pH of 6.0. The biopanning strategy also considered how the cell surface targets were presented as ECD proteins. We utilized both recombinant protein antigens with different tags (i.e. the Fc domain, Avitag with biotin, or poly-histidine), as well as on cells. The target antigens were biopanned in different conditions to sample distinct conformations. Moreover, cell-based biopanning was performed for membrane-bound targets expressed on either recombinant or endogenous cells. The biopanning process was tested with up to 42 out of 96 possible selections per protein target designed for the KingFisher machine. After three target rounds of biopanning, with a negative round, the final round was grown overnight without phage amplification and conditions to repress expression. These final cultures were used to produce DNA that served as the source for NGS amplicon processing.

### Selecting the paratope diversity from the biopanned VHO sequences

In each target biopanning campaign, more than 10 million sequences were produced, resulting in a large diverse pool of VHOs. The total DNA containing the diverse biopanned repertoire of VHOs was applied to a Telesis Bio BioXP™, equipped with their NGS amplicon library program. The MiSeq NGS reading technology was used to generate up to 25 million sequences, while the Nextgen NGS technology was used to generate more than 50 million sequences. Since handling such a large amount of data was a challenge, the NGS software program (PipeBio) was used to perform most of the processing that included merging paired-end sequence reads, aligning and translating the VHO amino acid framework, counting the enrichment of all the clones reads, identifying and grouping any CDR3 sequences with ≥90% similarity, and merging data between multiple project biopanning groups.

**Figure 3:**
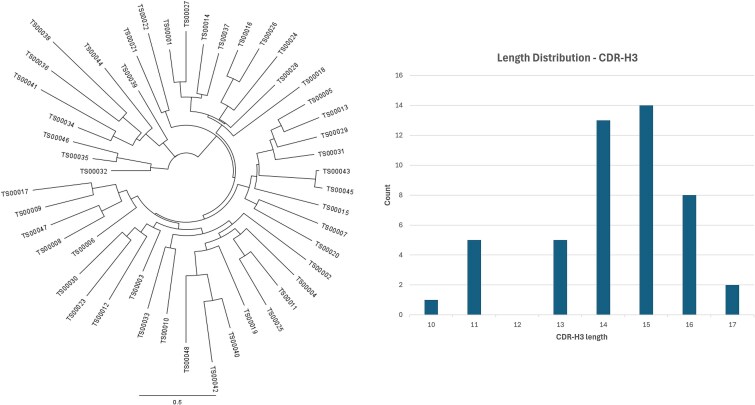
(A) Phylogenic tree representing the 48 candidates further characterized after all NGS processing, analysis, and filtering was applied, and Phylogenic tree created using geneious tree builder from geneious prime software; (B) summary of CDR-H3 lengths of the 48 VHO candidates.

To narrow down the best VHOs, the large NGS datasets were filtered to incorporate enrichment patterns of sequences based on the diverse biopanning groups, analyzed any posttranslational de-risking within library design areas (CDRs), and analyzed the patterns of hydrophobic and/or charged residues within CDR3. Finally, a set of sequences that could possibly lead to varying paratopes based on CDR3 sequence variations was selected. We typically focused on ≤100 VHOs for most of the projects to represent as much diversity as possible according to biopanning output and sequence analyses. Figure 3 is a phylogenic tree representing 48 VHO sequences from the target cMET. We provide further characterization data on some of these 48 candidates presented in Table 1.

**Table 1:** A summary of binding activities to different proteins and different cells

VHO	SEC	BLI with ECD Protein			ELISA with ECD Protein		Cancer Cell Line Binding						Bins
ID	%	human	cyno	mouse	human	cyno	AsPC-1 pancreas	BxPC3 pancreas	H292 lung	HCC827 lung	SNU-5 stomach	HEK293(−) kidney	#
VHO 03	95	X	X	X	X	X	X	X	X	X	X	-	1
VHO 05	86	X	X	X	X	X	X	X	X	X	X	-	1
VHO 06	93	X	X	X	X	X	X	X	X	X	X	-	1
VHO 07	83	X	X	X	X	X	X	X	X	X	X	-	2
VHO 08	68	X	X	X	X	X	X	X	X	X	X	-	2
VHO 09	90	X	X	X	X	X	X	X	X	X	X	-	2
VHO 04	89	X	X	X	X	X	X	X	X	X	X	-	3
VHO 10	45	X	X	-	X	X	X	X	X	X	X	X	4
VHO 11	70	X	X	-	X	X	-	-	-	X	X	X	5
VHO 12	86	X	X	X	X	-	X	X	X	X	X	-	6
VHO 13	62	X	X	X	X	-	X	X	X	X	X	-	6
VHO 14	82	X	X	X	X	-	X	X	X	X	X	-	7
VHO 15	69	X	X	X	X	-	-	-	-	X	X	-	8
VHO 16	98	X	X	X	X	X	X	-	-	X	X	-	9
VHO 17	82	X	X	X	X	X	X	-	-	X	X	-	9
VHO 18	67	X	X	X	X	X	X	-	-	X	X	-	9
VHO 19	69	X	X	X	X	X	-	-	-	X	X	-	10
VHO 20	72	X	X	X	X	X	-	-	-	X	X	-	10
VHO 21	80	X	X	X	X	X	-	-	-	X	X	-	10
VHO 22	94	X	X	X	X	X	-	-	-	X	X	-	10
VHO 23	85	X	X	X	X	X	-	-	-	X	X	X	11
VHO 24	81	X	X	X	X	X	-	-	-	X	X	X	11
VHO 25	59	X	X	-	-	-	-	-	-	X	X	-	12
VHO 26	93	X	X	X	-	-	X	X	X	X	X	-	13
VHO 27	61	-	X	X	X	X	X	X	-	X	X	X	14
VHO 28	74	X	X	X	-	-	-	-	-	-	-	-	15
Neg VHO	N/A	-	-	-	-	-	-	-	-	-	-	-	N/A
BM IgG	N/A	N/D	N/D	N/D	X	X	X	X	X	X	X	-	N/A

Each VHO is represented by their own unique ID. Positive binding is indicated by an “X” whereby having a greater response shift compared with a negative control VHO-fc and 5 times greater signal over the detection antibody at VHO concentrations of 5 nM. The VHOs in the chart named VHO 03, VHO 05, VHO 06, VHO 07, VHO 08 and VHO 09 are ones that internalized into MKN45 cells (see Figure 6). Bin # represents a different binding activity or epitope. The target for this project is cMet.

### Transient expression, purification, and evaluation of VHO-Fcs by SDS-PAGE and SEC

The Telesis Bio BioXP™ technology used an automated synthesis following NGS analysis to create a stable functional protein for each VHO. The BioXP™ cloning kit process assembled the VHO fragments of about 400 bp and spliced them individually into the expression plasmids that contained the human IgG Fc gene as a fusion tag. The cloning efficiency was >95%, which gave us the confidence to perform 3-mL scale transfections using the ExpiFectamine system (Gibco). After a 5-day transient expression incubation, the KingFisher system (Thermo Fisher) was used to automate the protein A or G affinity magnetic bead-based purification.

**Figure 4:**
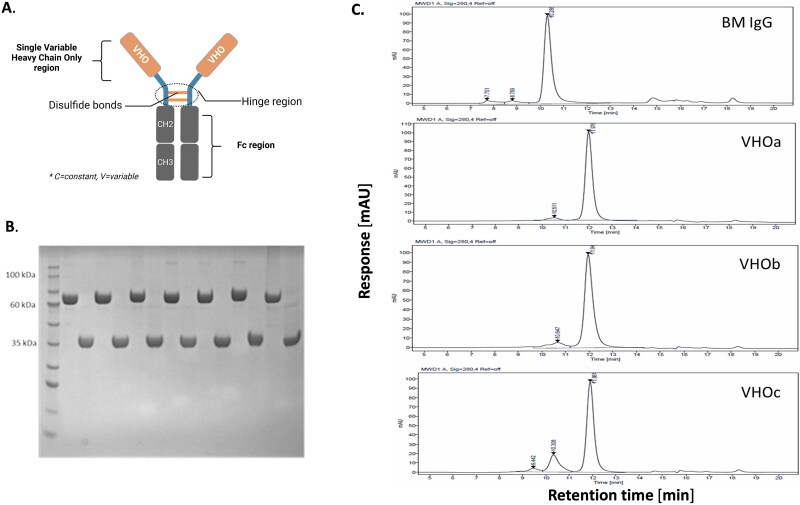
(A) The schematic diagram of a single VHO fused to a human IgG1 Fc region in a bivalent format; (B) SDS-PAGE showing single VHO-Fc purified proteins migrating at 75 kDa in an NR format, and 37 kDa in a reduced format, and (C) SEC analyses of three single VHO-Fc proteins along with a BM IgG protein at 150 kDa.

Typical yields range between 80 and 600 μg per VHO-Fc protein. Figure 4 shows a schematic representation of a single VHO-Fc protein and the SDS-PAGE results from ~1 μg of purified single VHO-Fc proteins. The single VHO-Fc proteins demonstrated the expected 75 kDa molecular weight size bands in a non-reduced (NR) format and 35 kDa for the reduced format.

SEC was used to assess the monodispersity of the single VHO-Fc. Figure 4C showed the SEC profiles of three VHO-Fcs in comparison to a BM IgG, with the *y*-axis response measured in mAU at 280 nm and the *x*-axis response measured in retention time in minutes. The usual range in percentage of the main peak area at an mAU at 280 nm for the VHO-Fc proteins was calculated to be between 80% and 90%.

### Determining activity bins from the paratope diversity of the VHO-Fcs

**Figure 5:**
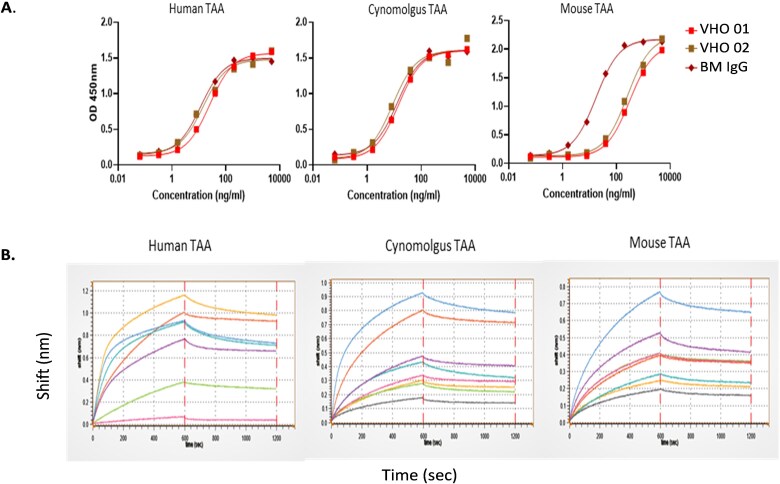
Assessment of species cross-reactivity from single VHO-Fcs; (A) by ELISA, VHO 01, VHO 02, and a BM IgG show binding to human, cynomolgus, and mouse proteins, and (B) by BLI, single VHO-Fcs bind to human, cynomolgus, and mouse proteins; the 6 VHO-Fcs are represented by the same-colored line for each VHO-Fc binding analysis to all 3 species proteins, and the negative control was represented by a pink line for human and black lines for cynomolgus and mouse proteins, and the BM IgG was represented by the orange line for human and pink lines for cynomolgus and mouse proteins.

Various target binding assays were conducted to determine the number of different activities represented within a VHO candidate pool. Protein binding assays were used for overall activity ranking and cross reactivity to human, murine, and cynomolgus monkey target proteins. Figure 5 displays the typical data captured for single VHO-Fcs that were cross-reactive to the TAA from human, cynomolgus, and mouse orthologs in both ELISA and BLI formats.

The TavoSelect platform prefers binding of the VHO-Fcs to be assessed by BLI, since this method mimicked the method of the biopanning process. Streptavidin sensor probes were used to capture biotinylated target protein (human TAA, cynomolgus TAA, and mouse TAA) during the loading step of a BLI assay. The VHO-Fc proteins and controls were added as analytes during the association step, and the subsequent wash with 1× PBS was used for the dissociation step. Using the Gator BLI system, the VHO-Fc candidates showed a range of affinities from 0.5 to 50 nM, with most of the candidates having a KD value between 1 and 10 nM. This affinity range is obtained from VHOs that have not been affinity matured. In Table 1 Bin # 1 and # 2 VHOs, represented by VHO 03 and VHO 07, respectively, are distinguished by binding activity differences to human target protein at pH 6 compared with pH 7 (data not shown). From at least 10 different targets, we obtained a 70%–99% protein binding success rate that correlated with the VHOs selected from the relative target biopanning and NGS campaign.

### VHOs with two different activities to a cell surface target

**Figure 6:**
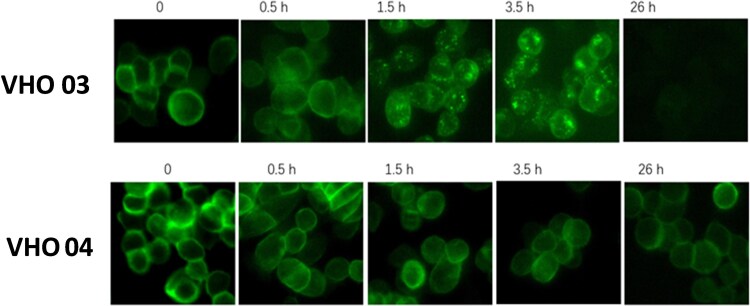
An internalization assay showing two single VHO-Fcs interacting with MKN45 cells; VHO 03 with a concentrated fluorescence signal inside the cells, and VHO 04 showed binding activity up to 26 h as indicated by the fluorescence on the cell’s surface.

To measure the epitope hit potential for applications in intracellular delivery, we evaluated the internalization of VHOs in a tumor cell line. Upon coupling of amine-reactive pHrodo™ dyes to the antibodies, the pHrodo™ assay was used to assess internalization. The amount of green fluorescence was used to measure the levels of VHO internalization into the cells. Both molecules showed a correlation in their binding interactions with the tumor cell line over various time points, measured in hours (Fig. 6). VHO 03 effectively caused internalization, with the level of green fluorescence increasing inside the tumor cell line up to 3.5 hours. While VHO 04 showed a higher level of green fluorescence on the surface of the tumor cell line, it did not show any sign of internalization signal out to 3.5 hours. VHO 04 was a stronger binder in comparison to VHO 03, as its cell surface fluorescent signal lasted for 26 hours. This internalization assay determined VHO 03 and VHO 04 of having different bin numbers or different epitopes in Table 1.

### Dual VHO show increased cell binding

**Figure 7:**
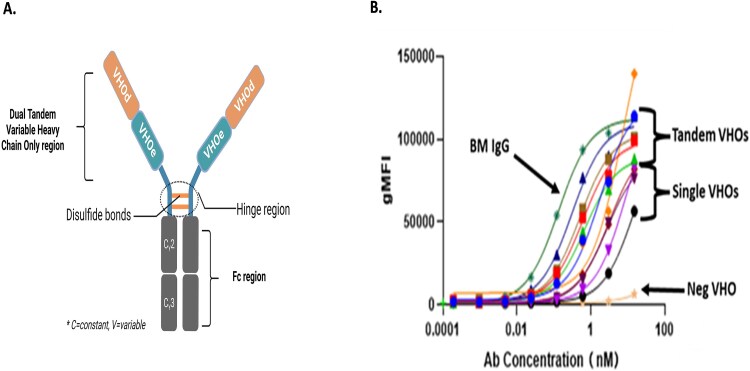
(A) A schematic representation of a dual VHO molecule, as a tandem format, where VHOe and VHOd recognize their own epitope; (B) dual tandem VHO-Fcs alongside related single VHO-Fcs, as well as a BM IgG (dark green line) and a negative control (opaque line) binding to a cancer cell line.

Tumor cell binding assays were conducted to determine whether the dual VHO-Fc molecules exhibited enhanced activity when compared with the single VHO-Fc format. We selected single VHOs containing different bin numbers or different epitopes for each, and engineered them as tandem formats on an Fc (Fig. 7A). Figure 7B shows tumor cell binding for the single VHO-Fcs and some combinations of the singles as tandem formatted VHO-Fcs along with a competitor BM IgG, as well as a negative control. There were single VHO combinations as tandems that bound less effectively than the single VHOs (data not shown). The dual tandem VHO-Fcs demonstrated superior overall binding to the tumor cells. The graph (Fig. 7B) shows that the dual tandem VHO-Fcs exhibited more potent EC50 values compared with the single VHO-Fcs. The dual VHO-Fcs were observed to have a similar efficacy range as the BM IgG.

### NFAT-luciferase reporter assay with U87MG cells

**Figure 8:**
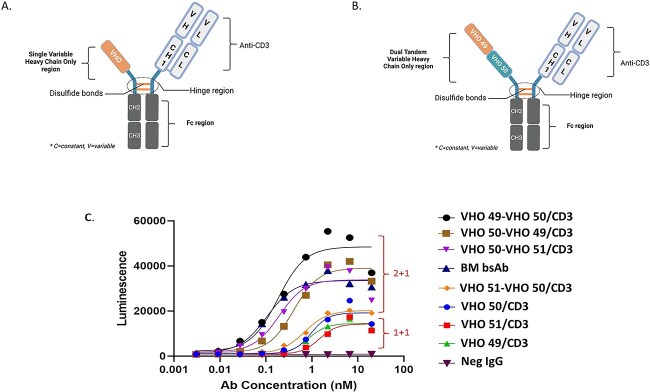
Schematic representations of 2 human Fc-based bispecifics each featuring one arm as a CD3 arm and (A) one single VHO arm and (B) the other a dual tandem VHO arm; (C) graph showing Jurkat NFATLuciferase activation assay against U87MG cells, and three of the single VHO/CD3 bispecific combinations depicted as the “1+1” group and the four dual tandem VHO/CD3 bispecific combinations depicted as the “2+1” group.

The TavoSelect platform engineers VHOs into custom scaffolds that deliver the most relevant MOA as possible. To illustrate the capabilities of multispecific antibodies (msAbs), a study was conducted where VHOs were paired with other functionalities, such as an anti-CD3 agonist antibody. Figure 8 showed that the schematic of this engineered bispecific format, where pairing of three VHOs (VHO 49, VHO 50, and VHO 51) in four different dual/tandem formats, along with the CD3 arm, was engineered as a fusion to a human Fc. The potencies of these combinations were assessed alongside a BM bispecific molecule. In this assay, the VHOs were also assessed in a single format, engineered along with the CD3 arm, both fused to the human Fc. The dual VHO format combination engineered with the CD3 arm as a bispecific antibody (bsAb) produced the best activity in this assay format. These results confirmed the expectations that certain VHO combinations played a role in cell functional activities. For example, not every dual VHO tandem could improve activity better than the single VHO bispecific format, as seen with the VHO 51-VHO 50 (N-term to C-term orientation), which did not improve activity such as the VHO 50-VHO 51 tandem orientation (Fig. 8C).

## Discussion

Although there is great value in using sdAb molecules for both therapeutic, research, and diagnostic purposes, there are few published reports that utilize a fully human synthetic phage display library [31] to date, and not much has been proven with such sdAb synthetic libraries for many disease targets. The traditional approach of generating monoclonal antibody (mAb) candidates for developable biotherapeutics from either naturally sourced (immunized or naïve) or synthetic antibodies is resource-intensive and lacks the ability to produce enough valuable candidates that maintain disease relevance. Although naturally sourced antibodies show high levels of target affinity and stability, they have limited ranges in antigen recognition, with hit identification often capturing at most several candidates. The synthetic antibody approach can yield more epitope diversity among its candidates, but they tend to be less stable and have lower affinity values when compared with naturally sourced antibodies. Despite these challenges, the TavoSelect VHO platform’s creative biopanning, NGS analysis, and specific screening methodologies can deliver diverse epitopes in less time with less resources.

We used the sequence of the human VH3-23 with a binary residue located in framework 1 that does not alter conformation to design the VHOs. As a result, most hits behaved well throughout the discovery process contrasting previous reports that mentioned or showed developability challenges with human heavy variable domain antibodies [32–35]. We are intrigued by the ability of our unique approach to generate well-behaved VHO-Fc candidates without having to perform framework engineering. The level of diversity along with patterns within each of the three CDRs could play some role in VHO stability [2,33]. Likewise, the NGS analysis after panning played a role in the selection of well-behaved VHO-Fc candidates.

The TavoSelect process successfully generated diverse VHO candidates that identified new conformational activation sites on target antigens. Delivering such epitope diversity within the early stages of therapeutic biologics molecule discovery can provide quality in the end for the development of leads. Such diverse leads can cover a diverse patient population of a particular disease [36]. To increase on-disease target specificity and reduce off-disease target reactivity, having more than one target presentation would be needed in the biopanning process too [37]. Having a multitude of paratopes can generate candidates for diverse epitopes providing the best chance of obtaining many developable candidates [38,39]. Even more so, having many hits permits the generation of dual epitope and dual target molecules as therapeutic leads. The TavoSelect VHO platform could provide hits for engineering lead multi-specific molecules with an efficiency seldom achieved by others.

The TavoSelect platform focused primarily on generating VHOs for immuno-oncology targets. To achieve this, we developed guidelines that included: biopanning target antigens under disease relevant conformations, environments, and in various formats [40]. During metastasis and tumor growth, the solid tumor microenvironment is acidic due to the tissue metabolism under hypoxic conditions. Therefore, biopanning experiments in such acidic conditions can expose more disease-specific epitopes. When conducting biopanning of antigens in different formats, pH selection can bias VHO binding toward the disease state target conformation at pH 6 over a normal state target conformation at pH 7–7.4. While many VHOs bound equally well to the target at both pH levels, some VHOs showed preferences for either pH 6 or pH 7–7.4. Since the number of selective VHOs was lower when only one pH was used, conducting biopanning in different pH values could lead to greater VHO paratope diversity. We also conducted biopanning using rodent and non-human primate antigen orthologs to generate the much-desired cross-reactivity while running *in vivo* testing of potency, efficacy, and safety.

Like other hit activity screening processes, NGS selection was preferred over binding activity screening assays after biopanning. NGS has proven to be a reliable replacement methodology for large-scale screening campaigns. Just like the traditional resource intensive ELISA, biopanning is a target binding assay too. Therefore, this is where an efficient process step such as NGS analysis can capture every possible candidate to obtain a broader diversity of the selected VHOs. The main goal of this sequence analysis was to evaluate as many paratopes as possible [39] and eliminate any candidates with posttranslational risks. Using all these diverse biopanning techniques mentioned, we can start to group or predict different activities within the VHO pool before any downstream characterizations are initiated.

Capturing NGS data from a biopanning campaign creates opportunities to apply machine learning. Our initial NGS analysis selects 32–100 candidates per campaign, with about 90% of them having different paratopes for processing protein. However, our initial strategy in candidate selection resulted in 50% of them having poor SEC profiles. With more projects completed, we were able to observe trends between VHO sequences and their SEC results. By applying these trends as filters into the NGS analysis, we have improved the SEC profiles of 70% of the VHOs. The minimal PTM risks found among the VHO generation projects were consistent with our initial design. As we continue to add to our VHO database, we expect to learn more about the relationships between sequences and physical protein behaviors. If applicable, learned trends will be incorporated into our NGS analysis scheme.

Careful selection of appropriate candidates from any antibody generation platform should be a priority, especially when using fully synthetic libraries and performing diverse biopannings. This combination of a highly diverse library and performing complex biopanning along with NGS generates massive numbers of VHO sequences. This poses a challenge in picking which VHOs to apply in hit to lead stage assessments. The analysis of sequences along with what biopanning situation the sequences came from has consistently produced VHO diversities from at least 10 projects. We observed with this triaging methodology that around 70% of the VHOs possessed correlative binding activities between biopanning and BLI binding assessments. The diverse paratopes within the selected pool of VHOs have also consistently been delivered on covering more epitope space per target than a traditional IgG. Performing binding assays to varied target presentations as both soluble protein and on the surface of more than one cell line has provided an even more confident assessment in picking through the VHOs in comparison to only a competitive approach as with epitope binning methods. VHO 3 of bin #1 had the best internalization activity, VHO 7 of bin #2 had a lower level of internalization (data not shown), and VHO 4 of bin #3 did not internalize. The many activity bins in Table 1 come from the application of the VHO scaffold library in conjunction with the diverse biopanning and NGS selection techniques.

Effective NGS result analysis that sorts and ranks millions of sequences down to less than 100 different VHO activities or epitopes, has allowed such VHOs to be placed with higher confidence into more therapeutic relevant assessments. For example, the internalization experiment, having more than one epitope between two VHOs, provided a way to prioritize candidates for either external or internal therapeutic engagement. This enabled a more efficient approach to developing antibody–drug conjugate (ADC) type therapy for oncology and inflammation.

NGS analysis allowed for *in silico* developability assessment. Avoiding residue sequence motif patterns for the most part played a role in picking candidates with behaved biophysical activity [41]. The monodispersed profiles shown in Fig. 4C are what we observe for most of the VHO-Fc candidates that do not have posttranslational risks such as N-link glycosylation, deamination, isomerization, oxidation, and hydrophobic patches especially in CDR3. VHOs representing an SEC profile of >80% monodispersity, see bottom most SEC profile in Fig. 4C, were chosen to be evaluated in downstream activity assays. These purified candidates with such SEC profiles did yield quality results as presented in the previous figures. While some VHOs had some precipitation during the neutralization step in the purification procedure, most VHOs remained stable for at least 2 years at 4°C.

The best VHO candidates with good protein purity and unique target activity were considered for lead optimization. Lead optimization usually consisted of building multi-specifics [42] with more than one VHO or in combination with other molecules, such as scFv, Fabs, VHHs, and/or other biologicals (Fig. 8). Alternatively, the VHO candidates can be used for selecting differentiated ADC, CAR-Ts, and antibody–oligonucleotide conjugates. To generate higher selectivity, the TavoSelect process could make tandem VHOs to engineer more than one activity into a single molecule quickly. We observed that two single VHO-Fcs that behaved unfavorably after purification could behave favorably when combined as a bi-specific after arm exchange. As seen in Figs 7 and 8, the avidity effects when combining two different VHOs caused an increase in activity toward the target. We have seen in other projects that when two VHOs were combined, the avidity effect could show up as an improved activity during an *in vivo* experiment. In other words, we created variants of VHOs that could be incorporated into distinct diagnostic and therapeutic modalities [24, 42].

A drug with a single mechanism of action may not be effective in treating a complex disease. In addition, high-potency therapeutics often have dose limiting side effects. Two main factors need to be considered to obtain potent therapeutic molecules: the therapeutic mechanism of action required to address the disease and the ability to efficiently manufacture the molecule. Therefore, engineering biologics with care would be necessary to achieve the best therapeutic index while enabling efficient CMC development process. Going forward, we aim to leverage the diversities provided by the TavoSelect VHO platform to meet these two main requirements.

## Abbreviations

Ab—antibody; ADC—antibody-drug conjugate; biopanning—biological panning; BM— benchmark; bsAb—bispecific antibody; CAR—chimeric antigen receptor; CH2—Constant Heavy Chain region 2; CH3—Constant Heavy Chain region 3; cyno—cynomolgus monkey; MOA—mechanism of action; BLI—biolayer interferometry; Bt—biotinylation; CDR—complementarity-determining region; CL—constant domain of light chain; EC50—half maximal effective concentration; ECD—extra-cellular domain; EF—effector function; ELISA—enzyme-linked immunosorbent assay; Fab—fragment antigen binding region; Fc—fragment crystallizable region; FcR—Fc receptor; h—hours; Ig—immunoglobulin; IMGT—International Immunogenetics Information System; kDa—kilo-Daltons; 2-MEA—2-mercaptoethylamine-HCl; MOA—mechanism of action; msAb—multi-specific antibody; NGS—next generation sequencing; Ni-NTA—Nickel-nitro acetic acid; OPD—o-phenylenediamine dihydrochloride; PBS—phosphate-buffered saline; PK—pharmacokinetics; SA—streptavidin; scFv—single chain fragment variable; sdAb—single domain antibody; SDS-PAGE—sodium dodecyl sulfate–polyacrylamide gel electrophoresis; SEC—size exclusion chromatography; TAA—tumor-associated antigen; T—target; TME—tumor microenvironment; mAU—measured absorbance units; Vh—variable heavy; Vk—variable kappa; VHO—variable heavy only; VLP—viral lipoparticles.

## Data Availability

The data that support the findings of this study are available on request from the corresponding author.

## Animal research

There was no animal research described in this manuscript.
